# Sex- and cell type-specific effects of dexmedetomidine on ferroptosis in neurons and microglia following traumatic brain injury in juvenile mice

**DOI:** 10.1007/s12035-025-05281-x

**Published:** 2025-11-06

**Authors:** Jie Fan, Stefanie Tasevski, Yara Mashal, Tia Atoui, Zahrah Naseer, Zhi Zhang

**Affiliations:** Department of Natural Sciences, College of Arts, Sciences, and Letters, University of Michigan-Dearborn, 4901 Evergreen Rd, Dearborn, MI 48128 USA

**Keywords:** TBI, Ferroptosis, Inflammation, Microglia, Neuron, Oxidative stress

## Abstract

Ferroptosis plays a key role in neuronal death and functional outcomes following traumatic brain injury (TBI). While TBI affects diverse cell types in injured brain regions, the sex- and cell type-specific responses to ferroptosis during the acute phase in the immature brain remain poorly understood. Dexmedetomidine (DEX), a selective alpha-2 adrenergic receptor agonist, has been shown to reduce inflammation and improve survival outcomes in TBI patients, but its sex- and cell type-specific effects on ferroptosis are unclear. This study investigated whether TBI induces sex- and cell type-specific ferroptotic responses and examined the effects of DEX during the acute post-injury phase in a juvenile mouse model. By concurrently isolating neurons and microglia from the same animals, we demonstrate that TBI prompts distinct sex- and cell type-specific ferroptosis responses, including differential regulation of genes involved in iron and lipid metabolism, oxidative stress, and proinflammatory pathways in neurons and microglia. DEX treatment significantly improved behavioral outcomes and reduced iron overload, lipid peroxidation, and neuroinflammation, thereby decreasing ferroptosis in both neurons and microglia. However, its effects were less prominent in neurons from female mice. Further analysis indicated that these sex- and cell type-specific responses to DEX may be partially due to differences in alpha-2 adrenergic receptor expression in neurons and microglia following TBI. Overall, our findings offer new insights into the mechanisms underlying sex- and cell type-specific responses to ferroptosis and DEX treatment, highlighting the broader implications for lipid metabolism, oxidative stress, and inflammation in the immature brain during the acute phase after TBI.

**Graphical Abstract:**

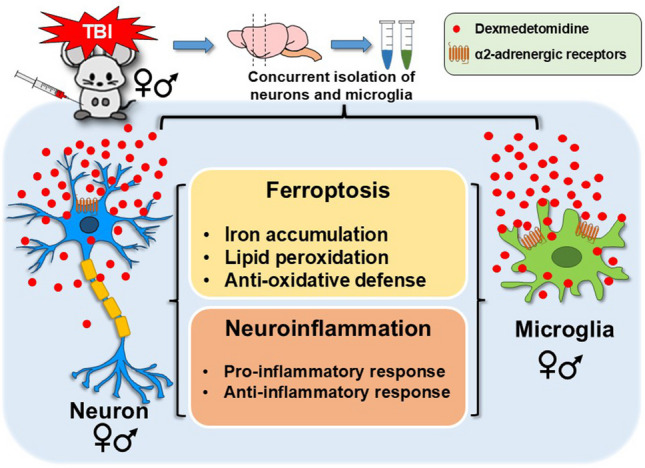

**Supplementary information:**

The online version contains supplementary material available at 10.1007/s12035-025-05281-x.

## Introduction

Traumatic brain injury (TBI) can cause immediate hemorrhage, while hemoglobin can be metabolized into ferrous/ferric iron, resulting in iron overload and cell ferroptosis at the site of injury [1]. Ferroptosis is a form of regulated, nonapoptotic cell death driven by redox imbalance and iron dependency. Key hallmarks of ferroptosis include accumulation of reactive oxygen species (ROS), iron deposition, irreparable lipid peroxidation, and membrane permeabilization. [2–5]. Through its impact on lipid metabolism, oxidative stress, and neuroinflammation, ferroptosis has been linked to neuronal death and impaired functional outcomes in adult TBI models [6–8], however, little is known about ferroptosis after TBI in immature brains.

The developing brain possesses distinct anatomical and physiological characteristics, such as ongoing synaptogenesis and myelination, which make it especially susceptible to injury [9]. The immature brains also show a greater susceptibility to oxidative stress [10], in part, due to the high content of unsaturated fatty acids, high demand of oxygen consumption, and high availability of redox-active iron [11]. Compounding this susceptibility, immature brains exhibit lower activities of key antioxidant enzymes, such as glutathione peroxidase (GPX), particularly GPX4 [12]. GPX4 uses glutathione (GSH) as a cofactor to reduce polyunsaturated fatty acid (PUFA) lipid peroxides (L-OOH) to lipid alcohols (L-OH) [13], and plays a central role in the prevention of lethal lipid oxidation [14]. Under pathological conditions, such as TBI, decreased GPX4 exacerbates reactive oxygen species (ROS) accumulation, catastrophic membrane damage and cell death [8, 15], highlighting the need to understand TBI-induced ferroptosis in the immature brain where iron imbalance and diminished antioxidant capacity are prevalent.

TBI impacts multiple cell types within injured brain regions, most notably neurons and microglia [16–20]. Neurons are rich in phospholipids containing iron and PUFA and are susceptible to ferroptosis, especially under pathological conditions [21]. Microglia, as the brain's resident immune cells, possess genomic machinery for ferroptotic processes [22], and is an important player in a cellular ferroptotic cascade [23]. Microglia have high iron storage capacities and are most sensitive to iron overload compared to neurons [24, 25]. Iron accumulation in microglia can not only induce their own death but also alter their phenotype, promoting production and release of proinflammatory cytokines [24]. Studies have shown that anti-inflammatory microglia are more susceptible to ferroptosis, compared to pro-inflammatory microglia [26], due to their lower content of inducible NO synthase (iNOS) [26, 27]. Moreover, microglia act as an initiator of ferroptosis in neurons through excessive production of pro-inflammatory factors, including IL-8 and IL-1β [24, 25]. Despite the growing knowledge of ferroptosis mechanisms, how neurons and microglia differentially experience ferroptosis after pediatric TBI, and how these responses are shaped by biological sex, remains poorly understood. It is critical to explore and compare the responses of neurons and microglia to ferroptosis after TBI because these cells fulfill distinct, essential roles in brain development, injury response, and recovery. Understanding cell type- and sex-specific dynamics of ferroptosis can illuminate mechanisms underlying diverse outcomes after pediatric TBI and inform development of targeted, more effective therapeutic interventions.

Ferroptosis has been implicated in many neurological disorders [28], yet no targeted therapeutics are currently approved to mitigate its effects [29]. Dexmedetomidine (DEX), a highly selective α2-adrenergic receptor (α2-AR) agonist, is approved by U.S. Food and Drug Administration (FDA) for intensive care unit (ICU) and procedural sedation, including patients with TBI [30]. DEX exhibits effects on multiple systems, including cardiovascular, cerebral hemodynamic, and metabolic effects [31]. Recent studies have demonstrated that DEX decreases inflammation, attenuates endoplasmic reticulum stress, reduces neuronal cell death, and mitigates cognitive dysfunction [32–34]. DEX increases the levels of GSH ratio, attenuates lipid peroxidation and inflammation in pediatric hyperoxia-induced brain injury [35]. Despite its use in pediatric populations [36], little is known about DEX’s effect on ferroptosis in neurons and microglia after TBI in immature brains. In this study, we investigated the sex- and cell type-specific effects of DEX on neuronal and microglial ferroptosis in a juvenile mouse model of TBI, aiming to advance understanding of how ferroptosis, via alterations in iron and lipid metabolism, oxidative stress, and neuroinflammatory pathways, contributes to TBI outcomes and how targeted interventions might ameliorate these effects.

## Materials and methods

### Animals

Male and female C57BL/6 mice (2–3 months of age; Jackson Laboratory, Bar Harbor, ME) were in-house bred. All the pups were delivered naturally and remained with their mother after birth until weaning. All animals were housed under ambient conditions (20–22 °C, 40–60% relative humidity, and a 12-h light/dark cycle). Necessary precautions were taken throughout the study to minimize pain and stress associated with experimental treatments. All experiments were conducted in accordance with the Guide for the Care and Use of Laboratory Animals, 8th edition (National Research Council, National Academies Press, 2011) and complied with the ARRIVE (Animal Research: Reporting of In Vivo Experiments) guidelines. The pre-specified interim analysis was performed in line with the principles of replacement, reduction, and refinement to minimize unnecessary use of experimental animals. Experimental procedures were approved by the Institutional Animal Care and Use Committee (IACUC) of the University of Michigan (Protocol number: PRO00010860).

### Impact acceleration model of TBI

The impact acceleration model represents mild TBI with diffuse axonal injury, which replicates the pathophysiology that is commonly seen in humans caused by falls [37–39]. On postnatal day 20–21 (P20-21), male (M) and female (F) animals (n = 72, 36 M/36F) from the same litter were randomized into Sham (n = 24, 12 M/12F), TBI + saline (n = 24, 12 M/12F) and TBI + DEX (n = 24, 12 M/12F) groups using a random number generator. Randomization was stratified by sex. The TBI animals underwent injury procedures as previously described [20, 40–43]. A detailed description of the TBI procedure using the custom-made apparatus has been provided in previous publications [41]. In brief, after fully anesthetized with 4% isoflurane, the animal was placed chest-down on an in-house-made TBI apparatus, and its head was directly in the path of the falling weight (30 g, 1.0 m above the head). The lab personnel pulled the pin, allowing the weight to fall vertically through the guide tube to strike the animal on the head in the midline around 2 mm behind the eyes. The animal rapidly underwent a 180° rotation, falling through a trapdoor and landing in the supine position on a cushion. The animal was removed immediately from the apparatus and placed in a clean warm cage. Sham animals were anesthetized with 4% isoflurane without TBI impact. All animals were closely monitored postoperatively with weight and health surveillance recordings, as per IACUC guidelines. No animals were excluded, and no animals died during the experiments.

### Dexmedetomidine administration

Dexdomitor (dexmedetomidine hydrochloride, concentration of 0.5 mg/mL) was purchased from MWI Animal Health (Fenton, MI, USA) and diluted in 0.9% sterile saline at 10 μg/mL concentration. Animals in the TBI + DEX group received the intraperitoneal injection (IP) of DEX (25 µg/kg) at 15 min post-injury [44]. The TBI + saline group received the same volume of saline at 15 min post-injury. The Sham group did not receive any treatment.

### Body weight measurement and behavioral testing

The mean elimination half-life of DEX is 2–3 h [45], therefore, the behavioral testing was performed at 6 h post DEX administration to minimize the interference of sedative effects. Mice from the Sham (n = 24, 12 M/12F), TBI + saline (n = 24, 12 M/12F) and TBI + DEX (n = 24, 12 M/12F) groups underwent body weight measurement and behavioral testing before injury (baseline) and at 6 h post-DEX treatment. The personnel were blinded to experimental groups. All animals underwent the testing in the following orders: the body weight, grip strength and Rotarod. There was a 20–30 min break after each test, so the animals could rest and recover.

#### Body weight

Body weight was measured before injury (baseline) and at 6 h post-DEX treatment. The changes in the body weight were calculated as: (Body weight)_change_ = (body weight)_6 h_ – (body weight)_baseline_.

#### Grip strength

Muscular strength was evaluated with a grip strength test using a grip strength meter (BIOSEB, FL, USA) [20]. In brief, the grip strength meter was positioned horizontally, and the animals were held by the tail and lowered towards the apparatus. The animals were allowed to grab the metal grid and were then pulled backwards in the horizontal plane. The force applied to the grid was recorded as the peak tension. Each animal underwent the grip strength test in three consecutive trials. The results were recorded and averaged for each animal. The change in the grip strength was calculated as: (grip strength) _change_ = (grip strength)_6 h_ – (grip strength) _baseline_.

#### Rotarod

Sensorimotor coordination, endurance, and fatigue resistance were evaluated with a touchscreen five-station accelerating Panlab RotaRod for mice (BIOSEB, FL, USA) as previously published [20, 46]. Each animal was situated on a stationary rod for 10 s, and the rod was then set in motion with an accelerating speed of 3–30 rpm. Each animal underwent three consecutive trials, and the maximal duration of each trial was 5 min. For each trial, if the animal fell before it reached the 5-min maximum test time, the trial ended at the time of the fall, and the latency to the fall was recorded. The latency to the first fall in three consecutive trials were recorded and averaged for each animal. The change in the latency to the first fall was calculated as: (Latency) _change_ = (latency)_6 h_ – (latency) _baseline_.

### Concurrent isolation of primary microglia and neurons

Concurrent isolation of microglia and neurons was performed as previously described [20, 43, 47]. In brief, animals were euthanized after completion of behavioral tests [Sham = 12 (6 M/6F); TBI + saline = 12 (6 M/6F); TBI + DEX = 12 (6 M/6F)]. Brains were harvested and rinsed in HBSS (Cat# 14,175,095, Thermo Fisher Scientific, MA, USA) on ice. Meninges were removed and the cortex tissues from the area of injury (approximately between bregma + 2 mm and bregma −1 mm) in the TBI mice, and the matching areas in the sham mice were micro-dissected as previously described [20, 41]. Brain tissues were transferred to HABG solutions [60 mL Hibernate A (Cat# HA, BrainBits, LLC, IL, USA), 1.2 mL B27 (Cat# 17,504,044, Thermo Fisher Scientific, MA, USA), 0.176 mL L-Glutamine (200 mM) (Cat# A2916801, Thermo Fisher Scientific, MA, USA)], and minced (~ 0.5 mm) on ice. Brain tissues were incubated in HABG solution for 8 min at 30ºC in a Boekel shaking incubator with a shaking speed of 90 rpm (Cole-Parmer, Vernon Hills, IL, USA). Tissues were transferred to papain solutions [12 mg papain (Cat# LS003119, Worthington Biochemical Corp. NJ, USA) per 6 mL HA minus calcium (Cat# HA-Ca; BrainBits, LLC, IL, USA), 0.015 mL L-Glutamine (200 mM)], and incubated for 30 min at 30ºC in a shaking incubator with a shaking speed of 90 rpm. Tissues were washed in HABG solution for 5 min at room temperature, triturated with a sterile pipette for 45 s, and sat at room temperature for 1 min. The supernatants were collected, and the trituration was repeated two times. The supernatants were combined and centrifuged in OptiPrep™ Density Gradient Medium (Cat# D1556, MilliporeSigma, MA, USA) at 800 xg for 15 min at 22ºC, and the fractions of neurons and microglia were collected as previously described [20, 47]. Cells were washed in HABG solutions and centrifuged at 200xg for 2 min at 22ºC. Supernatants were removed, and cells were washed in HBSS solution and centrifuged at 200xg for 2 min at 22ºC. Cell pellets were harvested for RNA isolation, while a small portion of the cells were used for purity measurement and ferroptosis evaluation. To ensure clarity, neurons isolated from male and female animals were labeled as “MN” and “FN,” respectively, while microglia from male and female animals were designated as “MM” and “FM.” These abbreviations were used throughout the following text.

### Evaluation of mitochondrial lipid peroxidation in neuronal and microglial cell ferroptosis

The purity of cells was evaluated as we previously published and the cell purity was calculated as the following: Cell purity = the number of NeuN positive (or IBA1 positive) cells/total number of cells × 100% [20]. To investigate the impact of TBI on ferroptosis, primary neurons and microglia were pooled from four animals per experimental group and were cultured in triplicate. Neurons (320 cells/mm^2^) and microglia (320 cell/mm^2^) were plated and cultured in the micro 8-well slide (Cat.No:81826; ibidi USA, Inc. WI, USA) in culture media [DMEM/F12 supplemented with 10% FBS (Cat# F4135; MilliporeSigma, MA, USA), 100 U/mL penicillin–streptomycin (Cat# 30–002-CI; Corning; NY, USA) and Gibco B27 Supplement (Cat# 17,504,044, Thermo Fisher Scientific, MA, USA)] in 5% CO_2_ at 37 °C. Cells were then incubated with BioTracker 633 Red Mitochondria Dye (Cat# SCT137; final concentration of 100 nM; MilliporeSigma, MA, USA) and BioTracker™ Mitochondrial Lipid Peroxide Live Cell Ferroptosis Indicator (Cat# SCT261; final concentration of 100 nM; MilliporeSigma, MA, USA) in culture media for 30 min before imaging. Images (40x, 5–7 images/replicate, 3 replicates per group) were acquired using a Nikon Plan Apo objective (20X/0.9) through the Nikon Eclipse TS2R fluorescent microscope (Nikon, NY, USA).

### RNA isolation and quantitative real-time polymerase chain reaction (qPCR)

Total RNA was extracted using TRIZOL (Cat# T9424; MilliporeSigma, MA, USA), according to manufacturer’s instructions. RNA samples were quantified using the Nanodrop ND-2000 Spectrophotometer (Thermo Fisher Scientific, MA, USA). Single-stranded complementary DNA (cDNA) was reverse transcribed from RNA using the High-Capacity cDNA Reverse Transcription Kit with RNase inhibitor (Cat# 4,387,406; Thermo Fisher Scientific, MA, USA). qPCR was performed with iTaq(tm) Universal SYBR(R) Green Supermix (Cat# 1,725,125; Bio-Rad, CA, USA) with CFX connect real-time PCR detection system (Bio-Rad, CA, USA). Amplification conditions included 30 s at 95 °C, 40 cycles at 95 °C for 5 s, and 60 °C for 30 s. Primers were custom designed (Table 1) and ordered from Integrated DNA Technology (Coralville, IA). The comparative threshold cycle (Ct) method was used to assess differential gene expressions. The male and female sham groups served as reference controls, and glyceraldehyde 3-phosphate dehydrogenase (GAPDH) was utilized as the housekeeping gene. For each sample, gene expression levels were first normalized to GAPDH expression (ΔCt) (Supplemental Fig. 1A-T). Subsequently, these ΔCt values were normalized to the mean ΔCt of the corresponding male or female sham group to calculate the ΔΔCt. The 2-ΔΔCt gave the relative fold changes in gene expression. The results from sham and treatment groups were averaged and compared.

**Table 1:** Primers for qPCR

Gene	Forward primer	Reverse Primer
*Ireb2*	TTGGTGGCATTGAGACAGAG	AGCATTGGATGACCCAGTTAG
*Tfrc*	TCCTGTCGCCCTATGTATCT	CGAAGCTTCAAGTTCTCCACTA
*Hmox1*	GTACACATCCAAGCCGAGAA	TGGTACAAGGAAGCCATCAC
*Ftl1*	GTGGATCTGTGTCTTGCTTCA	TGCGACTGGAGAGACTTGTA
*Fth1*	AGCTGGCATGGCAGAATATC	CTGCCTCAGTGACCAGTAAAG
*Gpx4*	CCCACTGTGGAAATGGATGA	ACGCAGCCGTTCTTATCAA
*xCT*	GCAGTCGCAGGACTGATTTA	GAAGAGGCAGGTGAAGGAAA
*Chac1*	GCTTGTTTGTCTGGATCACTTC	TGGGTAACAGTATGGGTCAAC
*Acsl4*	CACACACTTCGACTCACTAGC	GGCTGTCCTTCTTCCCAAAT
*Prdx3*	CTAGGGACTTCTTGATGGCTAAC	GGCAGGCTAAGGGAAAGAAT
*Gss*	TCTTTGGAGTGTGGGAATGG	GCACTAGAACCTGCTGGAAA
*NADH dehydrogenase*	GGTATCTGATGGAACGCGATAG	GGAACAGATGACCATAGCTGAG
*Ptgs2*	GAAGATTCCCTCCGGTGTTT	CCCTTCTCACTGGCTTATGTAG
*Tnf-α*	TCAGCCGATTTGCTATCTC ATA	AGTACTTGGGCAGATTGACCTC
*Il-1β*	GGTGTGTGACGTTCCCATTA	ATTGAGGTGGAGAGCTTTCAG
*iNOS*	GGAATCTTGGAGCGAGTTGT	CCTCTTGTCTTTGACCCAGTAG
*Il-6*	GTCTGTAGCTCATTCTGCTCTG	GAAGGCAACTGGATGGAAGT
*Tgf-β1*	GGTGGTATACTGAGACACCTTG	CCCAAGGAAAGGTAGGTGATAG
*Adra2a*	CCTGCCTGAGTGCTTAGAAA	GGGTGCTAGCCAAGAAAGAA
*Adra2b*	GAAGAGGAGGTGGAAGAATGTG	CTGCAAGGGTGGGTTGAATA
*Gapdh*	AACAGCAACTCCCACTCTTC	CCTGTTGCTGTAGCCGTATT

### Immunohistochemistry of brain tissues

Animals were euthanized after completion of behavioral tests [Sham = 12 (6 M/6F); TBI + saline = 12 (6 M/6F); TBI + DEX = 12 (6 M/6F)], and perfused transcardially with PBS. Brains were removed, postfixed in 10% formalin for 48 h, and then cryoprotected in 30% sucrose (in PBS). Coronal Sects. (20 µm, 1:6 series) were prepared on a cryostat (Leica Microsystems, IL, USA).

#### Co-staining of Perls' iron with neuron and microglia

Brain sections were immersed in freshly made Perls' solution (5% HCl and 5% potassium ferrocyanide) at room temperature for 30 min. The sections were then rinsed in deionized water and incubated for 15 min in 3,3’-diaminobenzidine (DAB) solution (Vector Laboratories, CA, USA). After wash, the sections were incubated overnight with Guinea pig anti-NeuN (a neuronal marker; 1:250; Cat# ABN90, MilliporeSigma, MA. USA) and rabbit anti-IBA1 (a microglial marker; 1:250; Cat# 019–19741, FUJIFILM Wako Chemicals USA, VA. USA). Sections were subsequently washed and incubated with fluorescent secondary antibodies (1:250; Life Technologies, MA, USA) for 2 h at room temperature. The slides were dried, and cover-slipped with fluorescent mounting medium with DAPI (Cat# F6057, MilliporeSigma, MA, USA).

#### Co-staining of 4-Hydroxynonenal (4-HNE) or malondialdehyde (MDA) with neuron and microglia

Brain sections were incubated overnight at 4 °C with mouse anti- 4-HNE (1:250; Cat# MA5-27,570, Thermo Fisher Scientific, MA. USA), or mouse anti-MDA (1:250; Cat# MA5-27,560, Thermo Fisher Scientific, MA. USA), Guinea pig anti-NeuN (1:250; Cat# ABN90, Sigma-Aldrich, Inc., MA. USA) and rabbit anti-IBA1 (Cat# 019–19741, FUJIFILM Wako Chemicals USA, VA. USA). Sections were subsequently washed and incubated with fluorescent secondary antibodies (1:250; Life Technologies, MA, U.S.A.) for 2 h at room temperature. The slides were dried, and cover-slipped with fluorescent mounting medium with DAPI (Cat# F6057, MilliporeSigma, MA, USA).

### Histology quantification

The histological quantification was performed in Sham (n = 12; 6 M/6F), TBI + saline (n = 12; 6 M/6F), and TBI + DEX (n = 12; 6 M/6F). Images (40X, 5 images/animal) were randomly acquired from the cortex area in the injured brain regions (approximately between bregma + 1 mm and bregma −0.5 mm) in TBI animals, or from the corresponding regions in the sham group using the Nikon Eclipse TS2R fluorescent microscope (Nikon, NY, USA). The camera settings were kept the same among groups. All slides and images were coded, and the analysis was performed with personnel blinded to the experiments.

The co-localization of NeuN^+^/iron^+^, IBA1^+^/iron^+^, NeuN^+^/4-HNE^+^, IBA1^+^/4-HNE^+^, NeuN^+^/MDA^+^, and IBA1^+^/MDA^+^ cells were analyzed using “Coloc 2” function in Fiji ImageJ (National Institutes of Health, NIH) followed the manufacturer’s instructions [42]. In brief, images were split into separate channels and were converted to 8-bit. Background subtraction was performed using Fiji ImageJ following the manufacturer’s instructions. The co-localization was analyzed using Kendall's Tau Rank Correlation test, and Costes' randomization was set at 10. Kendall’s Tau Rank Correlation test quantifies the spatial correlation between two image channels by assessing the agreement in the ranking of their pixel intensities. Specifically, it measures how consistently the relative order of intensities in one channel corresponds to that in the other. A value of 1 indicates perfect concordance, while a value of −1 signifies perfect discordance. To ensure the accuracy of analysis, data were manually checked with co-localization analysis as described previously [40, 41, 48, 49].

### Statistical analysis

All experimental data were included. Data were analyzed using GraphPad Prism 6 (Version 6.04; CA, USA) and IBM SPSS Statistics [Version: 28.0.1.1 (15)]. All data was presented as mean ± SEM. Normality of the data distribution was assessed using the D’Agostino and Pearson omnibus normality test. For comparisons between two groups, either a two-tailed Student’s t-test or a two-tailed Mann–Whitney U test was performed, depending on the distribution. For comparisons involving multiple groups or interactions between variables (e.g., sex, cell type, treatment), one-way, two-way, or three-way ANOVA was used as appropriate, followed by Tukey’s post hoc test to evaluate specific group differences. Statistical significance was set at *p* < 0.05 for all analyses.

For clarification, treatment effects were evaluated in neurons and microglia isolated separately from male and female mice across the Sham, TBI + saline, and TBI + DEX groups using one-way ANOVA followed by Tukey’s post hoc test. Cell type-specific effects were assessed by comparing neurons and microglia from each sex within the TBI + saline and TBI + DEX groups using a two-tailed Student’s t-test or Mann–Whitney U test. Sex-specific effects were evaluated by comparing neurons isolated from the male versus female mice, as well as microglia isolated from the male versus female mice, within the TBI + saline and TBI + DEX groups using a two-tailed Student’s t-test or Mann–Whitney U test.

To enhance data transparency, ΔCt values, representing target gene expression normalized to GAPDH, are presented in Supplemental Fig. 1 for the qPCR analysis. For imaging data analysis, the individual data points are provided in Supplemental Fig. 2.

## Results

### TBI induced ferroptosis responses in both neurons and microglia at the acute phase post-injury

We have previously demonstrated that TBI results in cell type-specific and sex-specific activation of ER stress pathways in neurons and microglia [20]. Here we evaluated the cell type-specific effects of TBI on ferroptosis, which is characterized by iron-dependent lipid peroxidation and subsequent membrane damage [50]. Primary neurons and microglia were concurrently isolated from the same brain tissues of individual animals, with cells pooled from four animals per experimental group. The purity of the isolated neuronal populations was approximately 93.4 ± 5.6%, while the purity of microglial populations was 99.7 ± 0.7%. Cells were cultured in triplicate and subsequently labeled with both a mitochondrial marker and a mitochondrial lipid peroxide live-cell ferroptosis indicator.

**Fig. 1:**
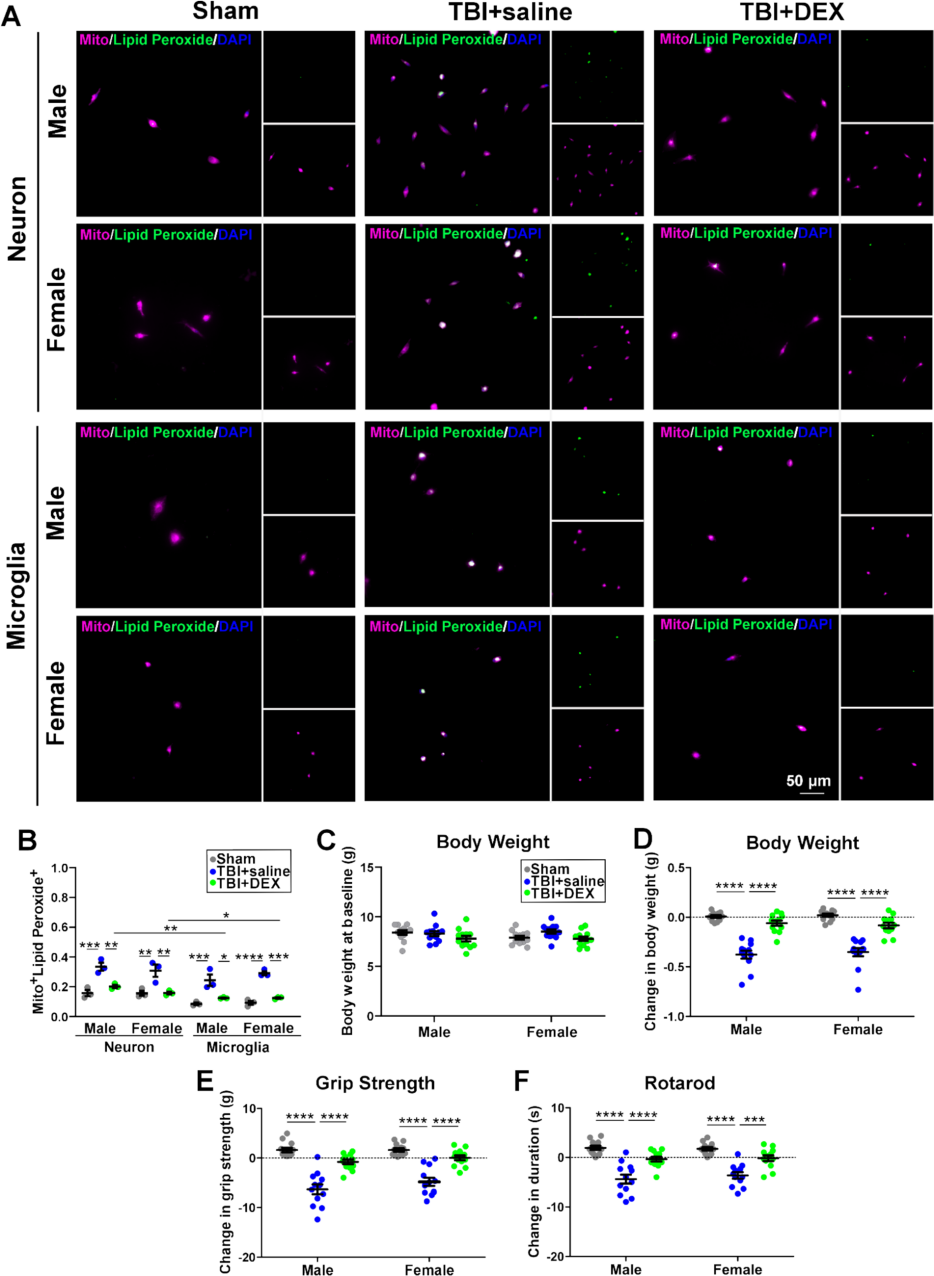
Cellular ferroptosis response, body weight and sensorimotor function evaluations. **A** The representative images of concurrently isolated neurons (upper panels) and microglia (lower panels) from male and female Sham, TBI + saline and TBI + DEX animals. Mitochondria (magenta); Mitochondrial lipid peroxide (a ferroptosis indicator; green); DAPI (blue). Scale bars: 50 µm. **B** Concurrently isolated primary neurons and microglia were pooled from 4 animals per group and cultured in 3 replicates. The co-localization of mitochondria and mitochondrial lipid peroxide significantly increased in the TBI + saline groups, compared with the sham and TBI + DEX groups in both males and females. **C** The baseline body weight before injury. **D** The body weight gain significantly decreased in the male and female TBI + saline groups, compared with the male and female Sham and TBI + DEX groups. **E** The grip strength significantly decreased in both male and female TBI + saline groups, compared with the male and female Sham and TBI + DEX groups. **F** In the Rotarod test, the latency to first fall significantly decreased in both male and female TBI + saline groups, compared with the male and female Sham and TBI + DEX groups. *, *p* < 0.05; **, *p* < 0.01; ***, *p* < 0.001; ****, *p* < 0.0001

Upon three-way ANOVA [cell type (neuron, microglia), sex (male, female), treatment (Sham, TBI + saline, TBI + DEX)], there were differences in the colocalization of mitochondria and mitochondrial lipid peroxide based on cell type [F_(1, 24)_ = 23.91, *p* < 0.0001] and treatment [F_(2, 24)_ = 77.09, *p* < 0.0001]. To evaluate the effects of treatment, we analyzed the colocalization of mitochondria and mitochondrial lipid peroxide in neurons and microglia isolated from male and female animals across the Sham, TBI + saline, and TBI + DEX groups. Upon one-way ANOVA (F = 17.62, *p* < 0.0001), mitochondrial lipid peroxide significantly increased in the neurons and microglia in both male and female the TBI + saline groups, compared with the Sham and TBI + DEX groups. To evaluate cell type-specific effects, we compared the colocalization of mitochondria and mitochondrial lipid peroxide between neurons and microglia within the TBI + saline and TBI + DEX groups for males and females separately. The mitochondrial lipid peroxide significantly decreased in microglia, compared with the neurons in the male [t_(4)_ = 6.91, *p* = 0.0023] and female [t_(4)_ = 3.22, *p* = 0.0323] TBI + DEX groups. There was no sex difference among groups (Fig. 1A, B; Supplemental Fig. 2A). Increased lipid peroxidation in mitochondria is a key factor in ferroptosis [51], which prompts the necessity for investigating the sex- and cell type-specific responses to ferroptosis, and evaluating the effects of DEX treatment at the acute phase post-TBI.

### DEX improved muscle strength and sensorimotor function at the acute phase post-injury

There was no significant difference in the baseline body weight among groups (Fig. 1C). To evaluate the effect of DEX on body weight, we compared the change in body weight at the baseline and at 6 h post-DEX treatment. Upon two-way ANOVA [sex (male, female), treatment (Sham, TBI + saline, TBI + DEX)], body weight significantly decreased in the male and female TBI + saline groups, compared with the male and female Sham and TBI + DEX groups based on treatment [F_(2, 66)_ = 89.80, *p* < 0.0001] (Fig. 1D).

To evaluate muscle strength and sensorimotor coordination, we compared the changes in grip strength and Rotarod performance at baseline and at 6 h post-DEX treatment. Upon two-way ANOVA [sex (male, female), treatment (Sham, TBI + saline, TBI + DEX)], grip strength significantly decreased in both male and female TBI + saline groups, compared with the male and female Sham and TBI + DEX groups based on treatment [F_(2, 66)_ = 66.76, *p* < 0.0001] (Fig. 1E). The latency to the first fall in the Rotarod tests significantly decreased in both male and female TBI + saline groups, compared with the male and female Sham and TBI + DEX groups based on treatment [F_(2, 66)_ = 49.27, *p* < 0.0001] (Fig. 1F).

### Sex- and cell type-specific effects of DEX on iron metabolism pathway activation

Ferroptosis is associated with iron accumulation and lipid peroxidation [52]. Iron-responsive element-binding protein 2 (IREB2), transferrin receptor (TFRC), heme oxygenase 1 (HMOX1/HO1), ferritin light chain 1 (FTL1) and ferritin heavy chain 1 (FTH1) are major regulators of iron absorption and metabolism. The mRNA expressions of IREB2, TFRC, HMOX1, FTL1 and FTH1 were analyzed in neurons and microglia in male and female Sham, TBI + saline and TBI + DEX animals. As described in the Methods section, neurons isolated from male and female animals are designated as “MN” and “FN,” respectively, while microglia from male and female animals are referred to as “MM” and “FM.”

**Fig. 2:**
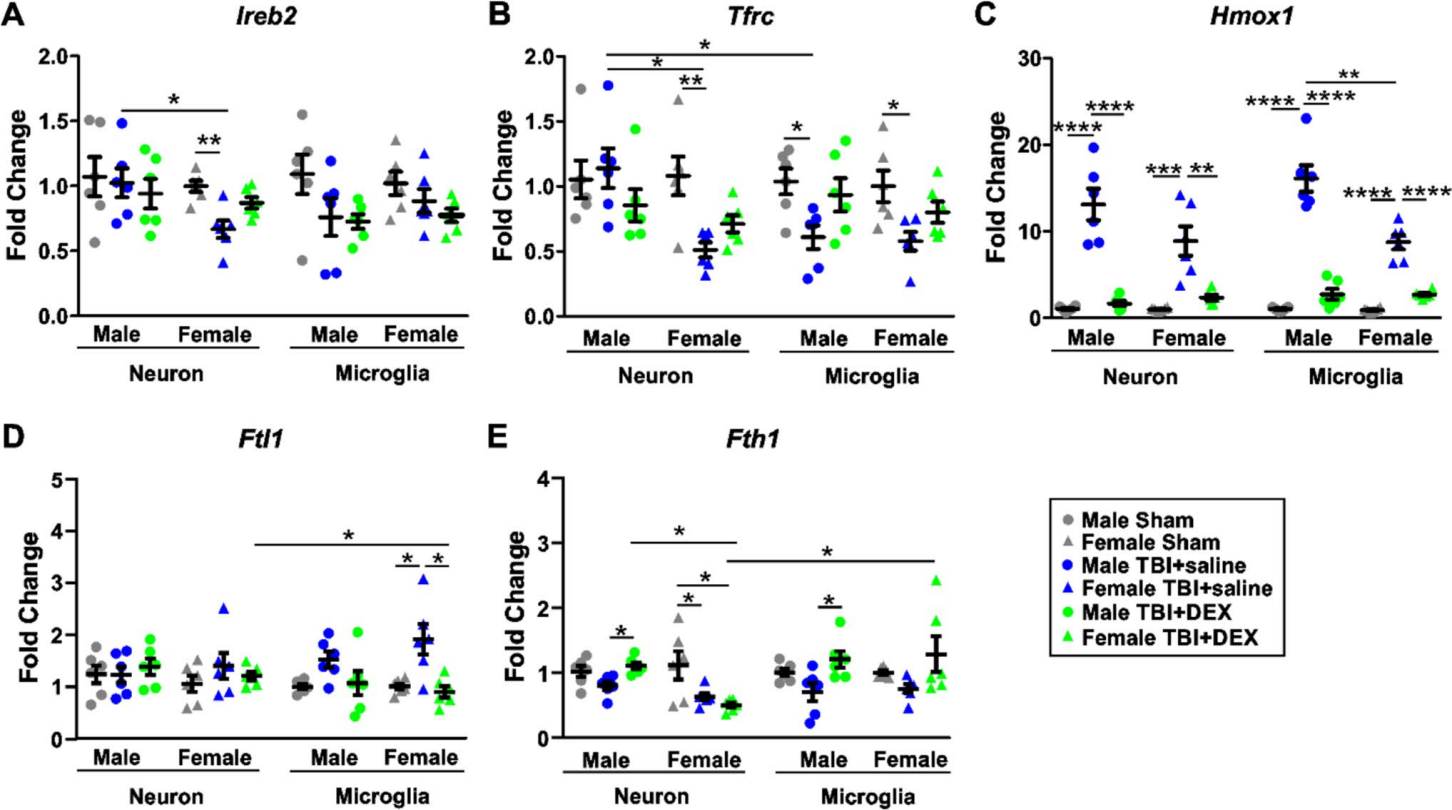
The mRNA expression of ferroptosis markers. The mRNA expression of *Ireb2* (**A**), *Tfrc* (**B**), *Hmox1* (**C**), *Ftl1* (**D**), and *Fth1* (**E**) in Sham, TBI + saline and TBI + DEX groups in both male and female mice. *, *p* < 0.05; **, *p* < 0.01; ***, *p* < 0.001; ****, *p *< 0.0001

Upon three-way ANOVA [cell type (neuron, microglia), sex (male, female), treatment (Sham, TBI + saline, TBI + DEX)], there were differences in *Ireb2* expression based on treatment [F_(2, 60)_ = 5.94, *p* = 0.004] (Fig. 2A). There were differences in *Tfrc* expression based on sex [F_(1, 60)_ = 5.92, *p* = 0.018] and treatment [F_(2, 60)_ = 9.13, *p* < 0.0001] (Fig. 2B). There were differences in *Hmox1* expression based on sex [F_(1, 60)_ = 12.461, *p* = 0.001], treatment [F_(2, 60)_ = 165.259, *p* < 0.0001], and sex*treatment [F_(2, 60)_ = 14.140, *p* < 0.0001] (Fig. 2C). There were differences in *Ftl1* expression based on treatment [F_(2, 60)_ = 7.881, *p* = 0.001] and cell type*treatment [F_(2, 60)_ = 4.826, *p* = 0.011] (Fig. 2D). There were differences in *Fth1* expressions based on treatment [F_(2, 60)_ = 8.230, *p* = 0.001] and cell type*treatment [F_(2, 60)_ = 4.804, *p* = 0.012] (Fig. 2E).

To evaluate the effects of treatment, we analyzed mRNA expression in neurons and microglia isolated from male and female animals across the Sham, TBI + saline, and TBI + DEX groups. Upon one-way ANOVA, the expression of *Ireb2* in FN (F = 9.77, *p* = 0.0019) significantly decreased in the TBI + saline group, compared with the Sham group (Fig. 2A). The expression of *Tfrc* in FN (F = 8.34, *p* = 0.0037), MM (F = 4.34, *p* = 0.0325) and FM (F = 5.07, *p* = 0.0208) significantly decreased in the TBI + saline group, compared with the Sham group (Fig. 2B). The expression of *Hmox1* in MN (F = 40.65, *p* < 0.0001), FN (F = 17.62, *p* = 0.0001), MM (F = 75.65, *p* < 0.0001), and FM (F = 67.96, *p* < 0.0001) significantly increased in the TBI + saline group, compared with the Sham and TBI + DEX groups (Fig. 2C). The expression of *Ftl1* in FM (F = 9.455, *p* = 0.0022) significantly increased in the TBI + saline group, compared with the Sham and TBI + DEX groups (Fig. 2D). The expression of *Fth1* in MN (F = 5.393, *p* = 0.0172) and MM (F = 4.876, *p* = 0.0234) significantly decreased in the TBI + saline group, compared with the TBI + DEX group; while the expression of *Fth1* in FN (F = 6.212, *p* = 0.0108) significantly decreased in the TBI + saline and TBI + DEX groups, compared with the Sham group (Fig. 2E).

To evaluate cell type-specific effects, we compared mRNA expression between neurons and microglia within the TBI + saline and TBI + DEX groups for males and females separately. *Tfrc* significantly decreased in MM compared with MN in the TBI + saline group [t_(10)_ = 3.00, *p* = 0.0134] (Fig. 2B). The expression of *Ftl1* significantly decreased in FM compared with FN in the TBI + DEX group [t_(10)_ = 2.311, *p* = 0.0434] (Fig. 2D). The expression of *Fth1* significantly increased in FM compared with FN in the TBI + DEX group [t_(10)_ = 2.830, *p* = 0.0178] (Fig. 2E).

To assess sex-specific effects, we compared mRNA expression between males and females in both neurons and microglia within the TBI + saline and TBI + DEX groups. *Ireb2* significantly decreased in FN compared with MN in the TBI + saline groups [t_(10)_ = 2.70, *p* = 0.0223] (Fig. 2A). *Tfrc* significantly decreased in FN compared with MN in the TBI + saline groups [t_(10)_ = 3.86, *p* = 0.0032] (Fig. 2B). *Hmox1* significantly increased in MM compared with the FM in the TBI + saline groups [t_(10)_ = 4.23, *p* = 0.0017] (Fig. 2C). The expression of *Fth1* significantly decreased in FN compared with MN in the TBI + DEX groups [t_(10)_ = 9.512, *p* < 0.0001] (Fig. 2E).

### DEX decreased iron accumulation in neurons and microglia

**Fig. 3:**
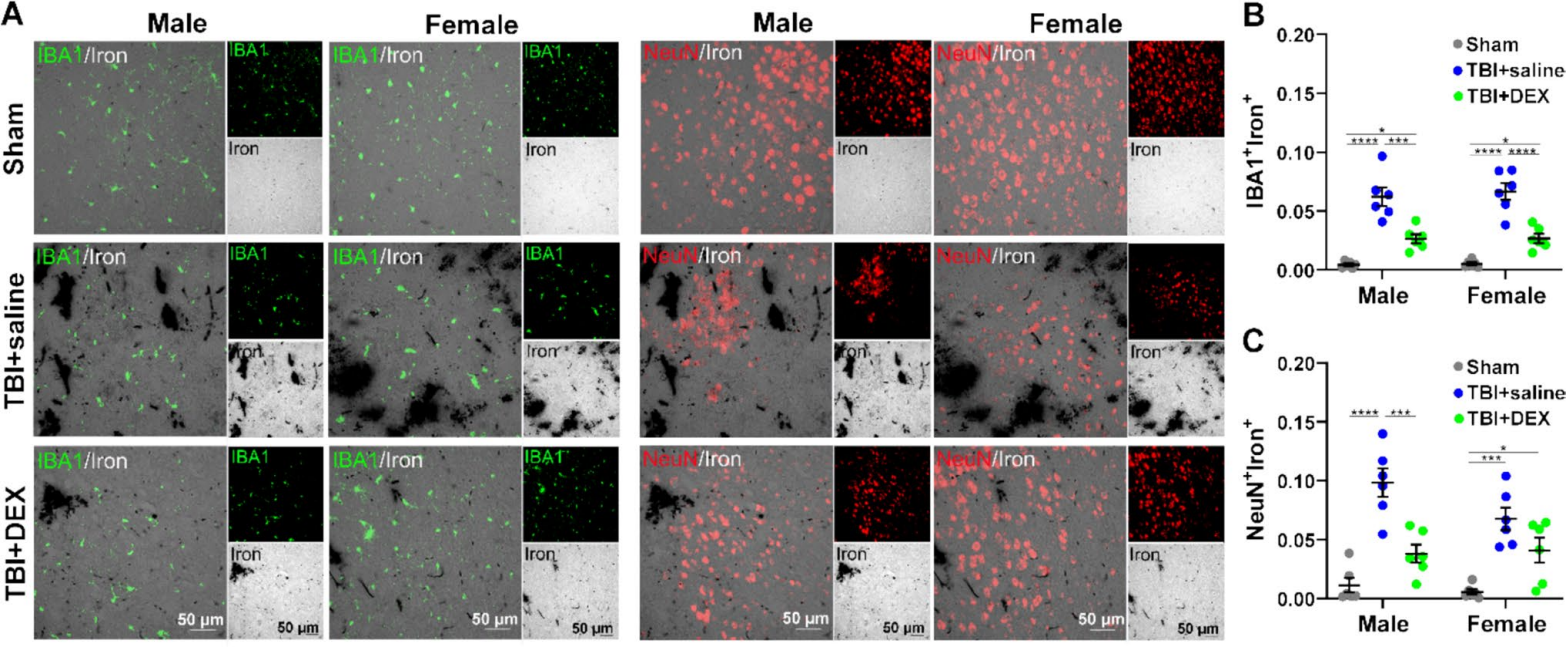
The co-localization of iron with neurons and microglia. **A** The representative images of iron (black), microglia (IBA1^+^, green) and neurons (NeuN^+^, red) colocalization. The left panels represent the co-localization of iron and microglia in the male and female Sham, TBI + saline and TBI + DEX groups. The right panels represent the co-localization of iron and neuron in the male and female Sham, TBI + saline and TBI + DEX groups. The scale bar: 50 µm. **B** The co-localization of iron and microglia (IBA1^+^Iron^+^) in the male and female Sham, TBI + saline and TBI + DEX groups. **C** The co-localization of iron and neurons (NeuN^+^Iron^+^) in the male and female Sham, TBI + saline and TBI + DEX groups. *, *p* < 0.05; ***, *p* < 0.001; ****, *p* < 0.0001

Intracellular iron metabolism and homeostasis is under delicate regulation and disruption of iron homeostasis increases free cellular iron contents. Therefore, we investigated the effects of DEX on intracellular iron accumulation in neurons and microglial cells. Upon two-way ANOVA [sex (male, female), treatment (Sham, TBI + saline, TBI + DEX)], there were differences in the expression of iron in microglia [F_(2, 30)_ = 73.46, *p* < 0.0001] and neurons [F_(2, 30)_ = 37.20, *p* < 0.0001] based on treatment (Fig. 3). To assess treatment effects, we compared intracellular iron accumulation in microglia and neurons isolated from male and female animals across the Sham, TBI + saline, and TBI + DEX groups. Upon one-way ANOVA, the accumulation of iron significantly increased in both MM (F_(2,15)_ = 32.09, *p *< 0.0001) and FM (F_(2,15)_ = 42.17, *p* < 0.0001) in the TBI + saline and TBI + DEX groups, compared with the Sham group. Moreover, the accumulation of iron significantly increased in both MM and FM in the TBI + saline group, compared with the TBI + DEX group (Fig. 3A, B). The accumulation of iron in MN (F_(2,15)_ = 24.67, *p* < 0.0001) significantly increased in the TBI + saline group, compared with the Sham and TBI + DEX groups. The accumulation of iron in the FN (F_(2,15)_ = 13.8, *p* = 0.0004) significantly increased in the TBI + saline and TBI + DEX groups, compared with the Sham group (Fig. 3A, C).

To assess cell type-specific effects, we compared iron accumulation between neurons and microglia within the TBI + saline and TBI + DEX groups for both males and females. The accumulation of iron significantly increased in MN compared with MM in the TBI + saline group [t_(10)_ = 2.506, *p* = 0.0311] **(**Fig. 3**)**.

To assess sex-specific effects, we compared iron accumulation between males and females in both neurons and microglia within the TBI + saline and TBI + DEX groups. There was no significant sex-specific difference detected **(**Fig. 3**)**.

### Sex- and cell type-specific effects of DEX on lipid metabolism and activation of oxidative stress pathways

Lipid metabolism and oxidative stress play critical roles in the regulation and execution of ferroptosis [52]. Dysregulation in lipid metabolic pathways can increase the abundance of peroxidizable lipids, priming cells for ferroptosis [53]. Ferroptosis is driven by the buildup of reactive oxygen species (ROS) and the breakdown of cellular antioxidant defenses. Oxidative stress, generated through both enzymatic and non-enzymatic reactions, leads to increased ROS, which readily attack vulnerable membrane lipids. Normally, antioxidants such as GSH and GPX4 work to neutralize lipid peroxides and protect the cell. However, when oxidative stress surpasses the cell’s antioxidant capacity due to increased ROS production, reduced antioxidant function, or depletion of GSH, lipid peroxides accumulate and promote ferroptosis. [54].

**Fig. 4:**
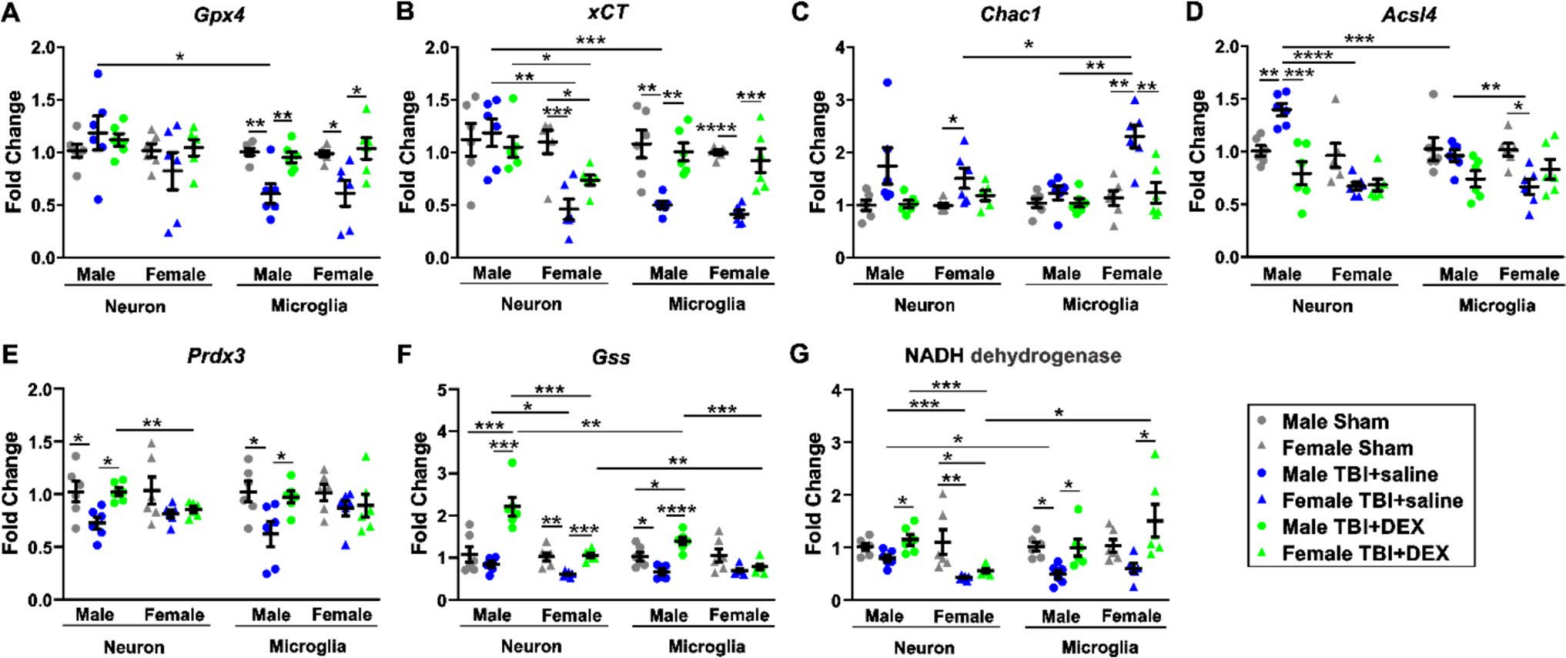
The mRNA expression of markers of lipid metabolism and oxidative stress. The mRNA expression of *Gpx4* (**A**), *xCT* (**B**), *Chac1* (**C**), *Acsl4* (**D**), *Prdx3* (**E**), *Gss* (**F**), and NADH dehydrogenase (**G**) in Sham, TBI + saline and TBI + DEX groups in both male and female mice. *, *p* < 0.05; **, *p* < 0.01; ***, *p* < 0.001; ****, *p* < 0.0001

GPX4, System Xc- (SLC7A11/xCT), glutathione specific gamma glutamylcyclotransferase 1 (CHAC1), acyl-CoA synthetase long-chain family member 4 (ACSL4), peroxiredoxin 3 (PRDX3), Glutathione Synthetase (GSS), and NADH dehydrogenase are involved in lipid metabolism and oxidative stress [50, 52, 55]. The mRNA expressions of GPX4, xCT, CHAC1, ACSL4, PDRX3, GSS and NADH dehydrogenase were analyzed in neurons and microglia in male and female Sham, TBI + saline and TBI + DEX animals. Upon three-way ANOVA [cell type (neuron, microglia), sex (male, female), treatment (Sham, TBI + saline, TBI + DEX)], there were significant differences in *Gpx4* expression based on cell type [F_(1, 60)_ = 8.80, *p* = 0.004], treatment [F_(2, 60)_ = 6.59, *p* = 0.003], and cell type*treatment [F_(2, 60)_ = 4.14, *p* = 0.021] (Fig. 4A). There were significant differences in *xCT* expression based on cell type [F_(1, 60)_ = 4.64, *p* = 0.035], sex [F_(1, 60)_ = 14.90, *p* < 0.0001], treatment [F_(2,60)_ = 20.28, *p* < 0.0001], cell type*sex [F_(1,60)_ = 5.68, *p* = 0.020], cell type*treatment [F_(2,60)_ = 5.13, *p* = 0.009], sex*treatment [F_(2,60)_ = 3.30, *p* = 0.044], and cell type*sex*treatment [F_(2,60)_ = 3.19, *p* = 0.048] (Fig. 4B). There was a significant difference in *Chac1* expression based on cell type [F_(1, 60)_ = 4.980, *p* = 0.029], sex [F_(1, 60)_ = 23.193, *p* < 0.0001], treatment [F_(2, 60)_ = 12.546, *p* < 0.0001], cell type*sex [F_(1, 60)_ = 4.980, *p* = 0.029], and sex*treatment [F_(2, 60)_ = 12.546, *p* < 0.0001] (Fig. 4C). There were significant differences in *Acsl4* expression based on sex [F_(1, 60)_ = 15.94, *p* < 0.0001], treatment [F_(2, 60)_ = 9.59, *p* < 0.0001], cell type*sex [F_(1, 60)_ = 5.94, *p* = 0.018], cell type*treatment [F_(2, 60)_ = 3.65, *p* = 0.032], and sex*treatment [F_(2, 60)_ = 12.98, *p* < 0.0001] (Fig. 4D). There were significant differences in *Prdx3* expression based on treatment [F_(2, 60)_ = 10.969, *p* < 0.0001] (Fig. 4E). There were significant differences in *Gss* expression based on cell type [F_(1, 60)_ = 9.133, *p* = 0.004], sex [F_(1, 60)_ = 25.240, *p* < 0.0001], treatment [F_(2, 60)_ = 33.098, *p* < 0.0001], cell type*sex [F_(1, 60)_ = 5.260, *p* = 0.025], cell type*treatment [F_(2, 60)_ = 6.825, *p* = 0.002], and sex*treatment [F_(2, 60)_ = 17.729, *p* < 0.0001] (Fig. 4F). There were significant differences in NADH dehydrogenase expression based on treatment [F_(2, 60)_ = 15.176, *p* < 0.0001], cell type*sex [F_(1, 60)_ = 6.636, *p* = 0.003], cell type*treatment [F_(2, 60)_ = 3.448, *p* = 0.038], and cell type*sex*treatment [F_(2, 60)_ = 4.481, *p* = 0.015] (Fig. 4G).

To assess treatment effects, we compared mRNA expression in neurons and microglia from males and females across the sham, TBI + saline, and TBI + DEX groups. Upon one-way ANOVA, the expression of *Gpx4* in MM (F = 10.88, *p* = 0.0012) and FM (F = 6.17, *p* = 0.0111) significantly decreased in the TBI + saline group, compared with the Sham and TBI + DEX groups (Fig. 4A). The expression of *xCT* in FN (F = 13.02, *p* = 0.0005) significantly decreased in the TBI + saline and TBI + DEX groups, compared with the Sham group. The expression of *xCT* in MM (F = 11.34, *p* = 0.0010) and FM (F = 20.78, *p* < 0.0001) significantly decreased in the TBI + saline group, compared with the Sham and TBI + DEX groups (Fig. 4B). The expression of *Chac1* in FN (F = 4.206, *p* = 0.0355) significantly increased in the TBI + saline group, compared with the sham group. The expression of *Chac1* in FM (F = 11.87, *p* = 0.0008) significantly increased in the TBI + saline group, compared with the Sham and TBI + DEX groups (Fig. 4C). The expression of *Acsl4* in MN (F = 15.37, *p* = 0.0002) significantly increased in the TBI + saline group, compared with the Sham and TBI + DEX groups. The expression of *Acsl4* in FM (F = 5.07, *p* = 0.0208) significantly decreased in the TBI + saline group, compared with the Sham group (Fig. 4D). The expression of *Prdx3* in MN (F = 6.224, *p* = 0.0108) and MM (F = 5.395, *p* = 0.0172) significantly decreased in the TBI + saline group, compared with the Sham and TBI + DEX groups (Fig. 4E). The expression of *Gss* in MN (F = 17.81, *p* = 0.0001) significantly increased in the TBI + Dex group, compared with the Sham and TBI + saline groups. *Gss* expression in FN (F = 14.74, *p* = 0.0003) significantly decreased in the TBI + saline group, compared with the Sham and TBI + DEX groups. *Gss* expression in MM (F = 17.90, *p* = 0.0001) significantly decreased in the TBI + saline and TBI + DEX groups, compared with the Sham group. Additionally, *Gss* significantly decreased in the TBI + saline group, compared with the TBI + DEX group (Fig. 4F). The expression of NADH dehydrogenase in MN (F = 5.107, *p* = 0.0203) and FM (F = 5.174, *p* = 0.0195) significantly decreased in the TBI + saline group, compared to the TBI + DEX group. NADH dehydrogenase in FN (F = 6.870, *p* = 0.0076) significantly decreased in the TBI + saline and TBI + DEX groups, compared with the Sham group. NADH dehydrogenase in MM (F = 6.968, *p* = 0.0072) significantly decreased in the TBI + saline group, compared with the Sham and TBI + DEX groups (Fig. 4G).

To evaluate cell type-specific effects, we further compared mRNA expression in neurons versus microglia within the TBI + saline and TBI + DEX groups for males and females, respectively. *Gpx4* significantly decreased in MM compared with MN in the TBI + saline group [t_(10)_ = 3.09, *p* = 0.0115] (Fig. 4A). *xCT* significantly decreased in MM compared with MN in the TBI + saline group [t_(10)_ = 4.99, *p* = 0.0005] (Fig. 4B). *Chac1* significantly increased in FM compared with FN in the TBI + saline group [t_(10)_ = 2.71, *p* = 0.0220] (Fig. 4C). *Acsl4* significantly increased in MN compared with MM in the TBI + saline group [t_(10)_ = 5.37, *p* = 0.0003] (Fig. 4D). *Gss* significantly increased in MN [t_(10)_ = 3.429, *p* = 0.0064] and FN [t_(10)_ = 3.175, *p* = 0.0099] compared with MM and FM in the TBI + DEX groups (Fig. 4F). NADH dehydrogenase significantly decreased in MM compared with MN in the TBI + saline group [t_(10)_ = 3.131, *p* = 0.0107]. NADH dehydrogenase significantly increased in FM compared with the FN in the TBI + DEX group [t_(10)_ = 3.131, *p* = 0.0107] (Fig. 4G).

To evaluate sex-specific differences, we further compared mRNA expression between males and females in both neurons and microglia within the TBI + saline and TBI + DEX groups. *xCT* significantly decreased in FN compared with MN in the TBI + saline [t_(10)_ = 4.44, *p* = 0.0013] and TBI + DEX groups [t_(10)_ = 2.89, *p* = 0.0162], respectively (Fig. 4B). *Chac1* significantly increased in FM compared with MM in the TBI + saline groups [t_(10)_ = 4.23, *p* = 0.0017] (Fig. 4C). *Acsl4* significantly increased in MN compared with FN in the TBI + saline groups [t_(10)_ = 10.12, p < 0.0001]. *Acsl4* significantly decreased in FM compared with MM in the TBI + saline groups [t_(10)_ = 3.29, *p* = 0.0082] (Fig. 4D). *Prdx3* significantly decreased in FN [t_(10)_ = 3.648, *p* = 0.0045] compared with MN in the TBI + DEX groups (Fig. 4E). *Gss* significantly decreased in FN compared with MN in the TBI + saline [t_(10)_ = 3.134, *p* = 0.0106] and TBI + DEX [t_(10)_ = 5.094, *p* = 0.0005] groups. *Gss* significantly decreased in FM compared with MM in the TBI + DEX groups [t_(10)_ = 5.476, *p* = 0.0003] (Fig. 4F). NADH dehydrogenase significantly decreased in FN compared with MN in the TBI + saline [t_(10)_ = 5.683, *p* = 0.0002] and TBI + DEX [t_(10)_ = 5.424, *p* = 0.0003] groups (Fig. 4G).

### DEX decreased lipid peroxidation in neurons and microglia

Ferroptosis is defined by the accumulation of iron-dependent lipid peroxides, which arise from the peroxidation of PUFAs in cellular membranes. The excessive build-up of these toxic lipid peroxides compromises membrane integrity and function, ultimately triggering ferroptotic cell death. Therefore, lipid peroxidation is not only a hallmark but also a central driver of ferroptosis, directly connecting membrane oxidative damage to ferroptosis [54]. 4-HNE and MDA, the reactive aldehydes produced by lipid peroxidation, increase during ferroptosis [52]. To evaluate the effects of DEX on lipid peroxidation, we measured 4-HNE and MDA expression in neurons and microglia.

**Fig. 5:**
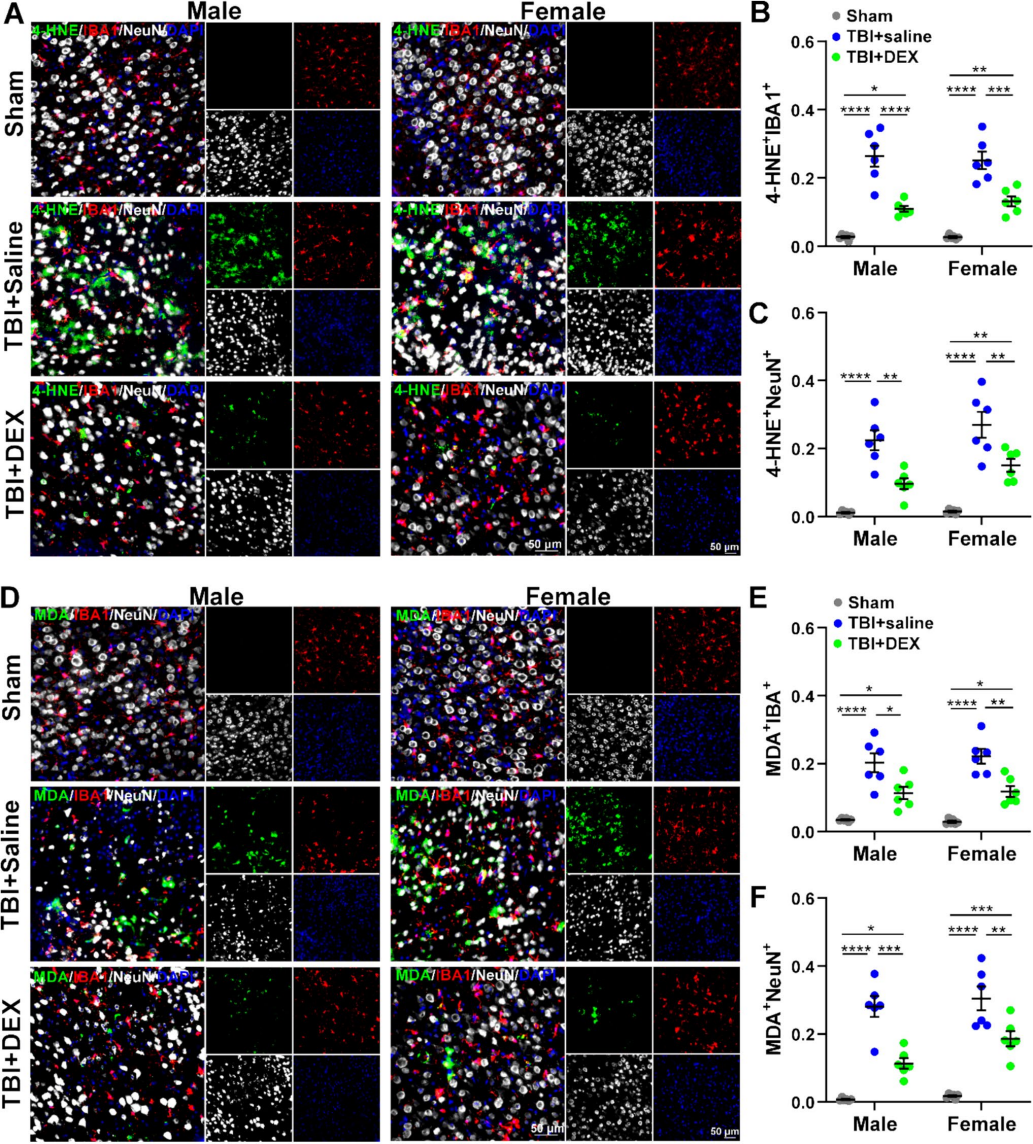
The expression of 4-HNE and MDA in neurons and microglia. **A** The representative images of 4-HNE (green), microglia (IBA1^+^, red) and neurons (NeuN^+^, white), and nuclei (DAPI^+^, blue) in male (left panels) and female (right panels) Sham, TBI + saline and TBI + DEX groups. The scale bar: 50 µm. **B** The co-localization of 4-HNE and microglia (4-HNE^+^IBA1^+^) in male and female Sham, TBI + saline and TBI + DEX groups. **C** The co-localization of 4-HNE and neurons (4-HNE^+^NeuN^+^) in male and female Sham, TBI + saline and TBI + DEX groups. **D** The representative images of MDA (green), microglia (IBA1^+^, red) and neurons (NeuN^+^, white), and nuclei (DAPI^+^, blue) in male (left panels) and female (right panels) Sham, TBI + saline and TBI + DEX groups. The scale bar: 50 µm. **E** The co-localization of MDA and microglia (MDA^+^IBA1^+^) in male and female Sham, TBI + saline and TBI + DEX groups. **F** The co-localization of MDA and neurons (MDA^+^NeuN^+^) in male and female Sham, TBI + saline and TBI + DEX groups. *, *p* < 0.05; **, *p* < 0.01; ***, *p* < 0.001; ****, *p* < 0.0001

Upon two-way ANOVA [sex (male, female), treatment (Sham, TBI + saline, TBI + DEX)], there were significant differences in 4-HNE expression in microglia [F_(2, 30)_ = 86.36, *p* < 0.0001] and neurons [F_(2, 30)_ = 55.9, *p* < 0.0001] based on treatment (Fig. 5 A-C). There were significant differences in MDA expression in microglia [F_(2, 30)_ = 53.54, *p* < 0.0001] and neurons [F_(2, 30)_ = 80.27, *p* < 0.0001] based on treatment (Fig. 5 D-F).

Upon one-way ANOVA, 4-HNE expressions in MM and FM (F = 24.75, *p* < 0.0001), as well as MN and FN (F = 23.40, *p* < 0.0001) significantly increased in the TBI + saline group, compared with the Sham and TBI + DEX groups. Moreover, 4-HNE expressions in MM, FM, and FN significantly increased in the TBI + DEX group, compared with the Sham group (Fig. 5A-C). MDA expressions in MM and FM (F = 21.65, *p* < 0.0001), as well as in MN and FN (F = 33.33, *p *< 0.0001) significantly elevated in the TBI + saline group compared to the Sham and TBI + DEX groups. Moreover, MDA expressions in MM, FM, MN and FN significantly increased in the TBI + DEX group, compared with the Sham group (Fig. 5D-F).

To assess cell type-specific effects, we further compared 4-HNE and MDA expression between neurons and microglia from both males and females within the TBI + saline and TBI + DEX groups. MDA significantly increased in FN compared with FM in the TBI + DEX group [t_(10)_ = 2.48, *p* = 0.0326] (Fig. 5).

To assess sex-specific effects, we compared 4-HNE and MDA expression between males and females in both neurons and microglia within the TBI + saline and TBI + DEX groups. There was no significant sex-specific difference detected (Fig. 5).

### DEX decreased pro-inflammatory responses in neurons and microglia

Neuroinflammation and ferroptosis are closely linked processes. Neuroinflammation disrupts iron metabolism, increases oxidative stress, and impairs antioxidant defenses, including the depletion of glutathione (GSH), thereby elevating the risk of ferroptosis. In turn, ferroptotic cell death amplifies inflammatory signaling, creating a vicious cycle that contributes to neural damage and the progression of neurological diseases [56]. Prostaglandin-endoperoxide synthase 2 (PTGS2), which encodes the enzyme cyclooxygenase-2 (COX-2), mediates ferroptosis by influencing arachidonic acid metabolism and inflammatory processes [57]. TNF-α enhances iron uptake and cytotoxicity [58], increases iron accumulation in microglia and shifts microglia towards proinflammatory polarization [59]. IL-1β increases the expression of ferroportin-1 in glial cells through the activation of p38-MAPK pathway, leading to excessive iron efflux and deposition in nerve cells and the environment [60].

**Fig. 6:**
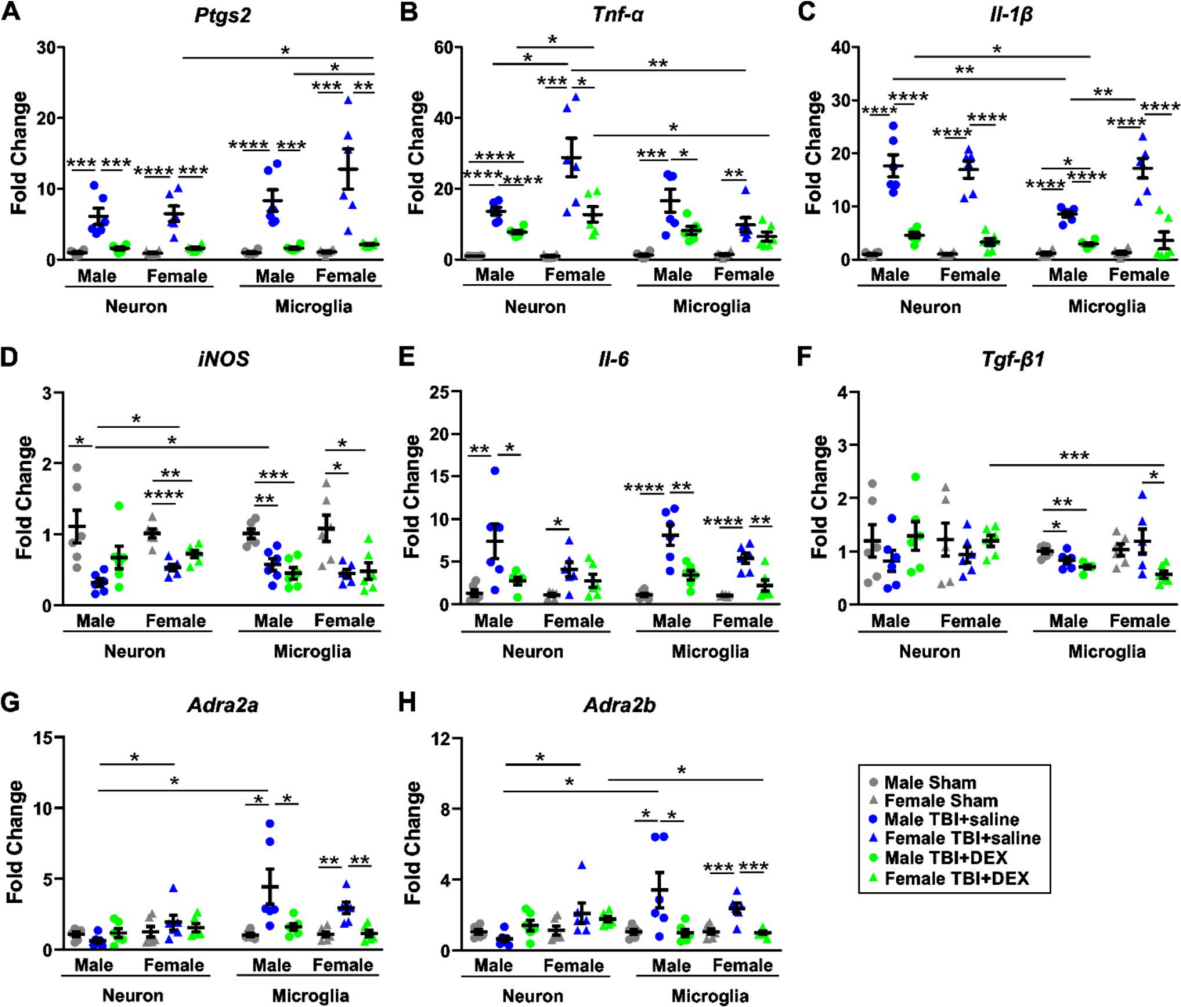
The mRNA expression of inflammatory markers and alpha 2-adrenergic receptors. The mRNA expression of *Ptgs2* (**A**), *Tnf-α* (**B**), *Il-1β* (**C**), *iNOS* (**D**), *Il-6* (**E**), *Tgf-β1* (**F**), *Adra2a* (**G**) and *Adra2b* (**H**) in Sham, TBI + saline and TBI + DEX groups in both male and female mice. *, *p* < 0.05; **, *p* < 0.01; ***, *p* < 0.001; ****, *p* < 0.0001

We further investigated the effects of DEX on the mRNA expression of PTGS2, TNF-α, IL-1β, iNOS, IL-6 and TGF-β1 in neurons and microglial cells. Upon three-way ANOVA [cell type (neuron, microglia), sex (male, female), treatment (Sham, TBI + saline, TBI + DEX)], there were significant differences in *Ptgs2* expression based on treatment [F_(2, 60)_ = 6.528, *p* = 0.003] (Fig. 6A). There were significant differences in *Tnf-α* expression based on cell type [F_(1, 60)_ = 8.35, *p* = 0.005], treatment [F_(2, 60)_ = 57.58, *p* < 0.0001], cell type*sex [F_(1, 60)_ = 15.13, *p* < 0.0001], cell type*treatment [F _(2, 60)_ = 3.99, *p* = 0.024], and cell type*sex*treatment [F_(2, 60)_ = 7.19, *p* = 0.002] (Fig. 6 B). There were significant differences in *Il-1β* expression based on cell type [F_(1, 60)_ = 6.84, p = 0.011], treatment [F_(2, 60)_ = 188.58, p < 0.0001], cell type*sex [F_(1, 60)_ = 9.27, *p* = 0.003], cell type*treatment [F_(2, 60)_ = 5.07, *p* = 0.009], sex*treatment [F_(2, 60)_ = 4.70, *p* = 0.013], and cell type*sex*treatment [F_(2, 60)_ = 5.12, *p* = 0.009] (Fig. 6C). There were significant differences in *iNOS* expressions based on treatment [F_(2, 60)_ = 28.998, *p* < 0.0001] (Fig. 6D). There were significant differences in *Il-6* expression based on sex [F_(1, 60)_ = 28.446, *p* = 0.012], treatment [F_(2, 60)_ = 39.184, *p* < 0.0001], and sex*treatment [F_(2, 60)_ = 3.414, *p* = 0.039] (Fig. 6E). There were significant differences in *Tgf-β1* expression based on cell type [F_(1, 60)_ = 4.424, *p* = 0.040] and cell type*treatment [F_(2, 60)_ = 4.021, *p* = 0.023] (Fig. 6F).

To evaluate the effects of treatment, we analyzed mRNA expression in neurons and microglia from males and females across the Sham, TBI + saline, and TBI + DEX groups. Upon one-way ANOVA, the expression of *Ptgs2* in MN (F = 8.315, *p* = 0.0037), FN (F = 22.35, *p* < 0.0001), MM (F = 24.28, *p* < 0.0001), and FM (F = 15.53, *p* = 0.0002) significantly increased in the TBI + saline group, compared with the Sham and TBI + DEX groups (Fig. 6A). The expression of *Tnf-α* in MN (F = 84.94, *p* < 0.0001) significantly increased in the TBI + saline and TBI + DEX groups, compared with the Sham group. Moreover, the expression of *Tnf-α* in MN significantly increased in the TBI + saline group, compared with the TBI + DEX group. The expression of *Tnf-α* in FN (F = 17.00, *p* = 0.0001) and MM (F = 14.94, *p* = 0.0003) significantly increased in the TBI + saline group, compared with the Sham and TBI + DEX groups. The expression of *Tnf-α* in FM (F = 8.97, *p* = 0.0027) significantly increased in the TBI + saline group, compared with the Sham group (Fig. 6B). The expression of *Il-1β* in MN (F = 50.07, *p* < 0.0001), FN (F = 73.23, *p* < 0.0001), and FM (F = 36.39, *p* < 0.0001) significantly increased in the TBI + saline group, compared with the Sham and TBI + DEX groups. The expression of *Il-1β* in MM (F = 111.50, *p* < 0.0001) significantly increased in the TBI + saline and TBI + DEX groups, compared with the Sham group. Moreover, the expression of *Il-1β* in MM significantly increased in the TBI + saline group, compared with the TBI + DEX group (Fig. 6C). *iNOS* in MN (F = 5.824, *p* = 0.0134) significantly decreased in the TBI + saline group, compared with the Sham group. *iNOS* in FN (F = 20.15, *p* < 0.0001), MM (F = 14.14, *p* = 0.0004), and FM (F = 7.439, *p* = 0.0057) significantly decreased in the TBI + saline and TBI + DEX groups, compared with the Sham group (Fig. 6D). *Il-6* in MN (F = 6.890, *p* = 0.0075), MM (F = 22.04, *p* < 0.0001), and FM (F = 19.42, *p* < 0.0001) significantly increased in the TBI + saline group, compared with the Sham and TBI + DEX groups. *Il-6* in FN (F = 4.804, *p* = 0.0244) significantly increased in the TBI + saline group, compared with the Sham group (Fig. 6E). *Tgf-β1* in MM (F = 10.23, *p* = 0.0016) significantly decreased in the TBI + saline and TBI + DEX groups, compared with the Sham group. *Tgf-β1* in FM (F = 4.680, *p* = 0.0263) significantly decreased in the TBI + DEX group, compared with the TBI + saline group (Fig. 6F).

To further investigate cell type-specific effects, we compared mRNA expression between neurons and microglia within the TBI + saline and TBI + DEX groups for both sexes. *Ptgs2* significantly increased in FM compared with FN in the TBI + DEX group [t_(10)_ = 2.88, *p* = 0.0165] (Fig. 6A). *Tnf-α* significantly decreased in FM compared with FN in the TBI + saline [t_(10)_ = 3.27, *p* = 0.0085] and TBI + DEX groups [t_(10)_ = 2.46, *p* = 0.0334] (Fig. 6B). *Il-1β* significantly decreased in MM compared with MN in the TBI + saline [t_(10)_ = 4.25, *p* = 0.0017] and TBI + DEX groups [t_(10)_ = 2.95, *p* = 0.0145] (Fig. 6C). *iNOS* significantly decreased in MN compared with MM in the TBI + saline group [t_(10)_ = 2.467, *p* = 0.0333] (Fig. 6D). *Tgf-β1* significantly decreased in FM compared with FN in the TBI + DEX group [t_(10)_ = 4.901, *p* = 0.0006] (Fig. 6F).

To investigate sex-specific differences, we compared mRNA expression between males and females in neurons and microglia within the TBI + saline and TBI + DEX groups. *Ptgs2* significantly increased in FM compared with MM in the TBI + Dex group [t_(10)_ = 3.591, *p* = 0.0049] (Fig. 6A). *Tnf-α* significantly increased in FN compared with MN in the TBI + saline [t_(10)_ = 2.73, *p* = 0.0212] and TBI + DEX groups [t_(10)_ = 2.25, *p* = 0.0486] (Fig. 6B). *Il-1β* significantly increased in FM compared with MM in the TBI + saline group [t_(10)_ = 4.50, *p* = 0.0011] (Fig. 6C). *iNOS* significantly decreased in MN compared with FN in the TBI + saline group [t_(10)_ = 3.062, *p* = 0.0120] (Fig. 6D).

### Sex and cell type-specific effects of DEX on alpha-2 adrenergic receptor expression at the acute phase post-injury

DEX acts as an agonist of alpha 2-adrenergic receptors [61]. Neurons and microglia may express different levels or subtypes of alpha 2-adrenergic receptors. The cellular response to DEX depends on the presence and density of its target receptor. Increasing evidence suggests that the expression of alpha 2-adrenergic receptors varies between sexes because of genetic, epigenetic, and hormonal factors [62]. These differences may affect how males and females respond to DEX.

To investigate the underlying mechanisms of the sex and cell type-specific effects of DEX, we measured the expression of alpha-2A adrenergic receptor (ADRA2A) and alpha-2B adrenergic receptor (ADRA2B) in male and female neurons and microglia. Upon three-way ANOVA [cell type (neuron, microglia), sex (male, female), treatment (Sham, TBI + saline, TBI + DEX)], there were significant differences in *Adra2a* expression based on cell type [F_(1, 60)_ = 8.606, *p* = 0.005], treatment [F_(2, 60)_ = 10.286, *p* < 0.0001], cell type*sex [F_(1, 60)_ = 5.730, *p* = 0.020], and cell type*treatment [F_(2, 60)_ = 9.831, *p* < 0.0001] (Fig. 6G). There were significant differences in *Adra2b* expression based on treatment [F_(2, 60)_ = 8.947, *p* < 0.0001], cell type*sex [F_(1, 60)_ = 4.993, *p* = 0.029], and cell type*treatment [F_(2, 60)_ = 8.484, *p* = 0.001] (Fig. 6H).

To assess treatment effects, we compared mRNA expression in neurons and microglia from males and females across the sham, TBI + saline, and TBI + DEX groups. Upon one-way ANOVA, the expression of *Adra2a* in MM (F = 6.316, *p* = 0.0102) and FM (F = 13.39, *p* = 0.0005) significantly increased in the TBI + saline group, compared with the Sham and TBI + DEX groups (Fig. 6G). The expression of *Adra2b* in MM (F = 5.467, *p* = 0.0165) and FM (F = 16.07, *p* = 0.0002) significantly increased in the TBI + saline group, compared with the Sham and TBI + DEX groups (Fig. 6H).

To evaluate cell type-specific differences, we further compared mRNA expression between neurons and microglia within the TBI + saline and TBI + DEX groups in males and females. *Adra2a* significantly increased MM compared with MN in the TBI + saline group [t_(10)_ = 3.076, *p* = 0.0117] (Fig. 6G). *Adra2b* significantly increased in MM compared with MN in the TBI + saline group [t_(10)_ = 2.749, *p* = 0.0205]. *Adra2b* significantly increased in FM compared with FN in the TBI + DEX group [t_(10)_ = 4.420, *p* = 0.0013] (Fig. 6H).

To investigate sex-specific effects, we compared mRNA expression between males and females in neurons and microglia within the TBI + saline and TBI + DEX groups. *Adra2a* significantly increased in FN compared with MN in the TBI + saline groups [t_(10)_ = 2.392, *p* = 0.0378] (Fig. 6G). *Adra2b* significantly increased in FN compared with MN in the TBI + saline groups [t_(10)_ = 2.465, *p* = 0.0334] (Fig. 6H).

## Discussion

Ferroptosis plays an important role in the long-term consequences of neurodegeneration and neurological impairment following TBI. Ferroptosis has biochemical features, including iron accumulation, imbalanced glutathione metabolism and lipid peroxidation [3]. In the present study, we have demonstrated that neurons and microglia exhibit different responses to ferroptosis, while DEX has cell type- and sex-specific effects on the ferroptosis at the acute phase post-injury in the immature brains.

Intracellular iron levels are tightly controlled through processes of uptake, export, storage, and utilization [63]. The IREB2 gene encodes iron regulatory protein 2 (IRP2), and the activation of IRP2 can induce accumulation of cellular iron and promote ferroptosis [64]. IRP2 also plays an important role in post-transcriptional regulation of iron metabolism-related proteins, such as TFRC [65]. TFRC regulates cellular iron uptake, while inhibiting TFR1 can efficiently block ferroptosis [66]. In the present study, we have shown that the mRNA expressions of IREB2 and TFRC significantly decreased in the neurons isolated from the female TBI + saline group but did not change in the male TBI + saline group. Moreover, the mRNA expressions of TFRC significantly decreased in microglia isolated from both male and female TBI + saline groups. Microglia play a role in regulating iron homeostasis [67], as iron accumulation in activated microglia triggers pro-inflammatory cytokine release and exacerbates neuronal iron deposition [68]. Thus, the reductions in IREB2 and TFRC may represent an acute compensatory response by cells to increased iron exposure following injury. HMOX1 is the rate-limiting enzyme that catalyzes heme into equimolar quantities of iron, biliverdin, and carbon monoxide, and plays a dual role in ferroptosis [52]. Elevated HMOX1 mRNA expression and increased iron accumulation in neurons and microglia point to enhanced ferroptosis during the acute phase following injury. However, DEX treatment significantly attenuated this process. Notably, DEX reduced microglial iron accumulation in both males and females, while its effect on neuronal iron accumulation was observed only in males and not in females. This finding suggests sex-specific differences in the protective effects of DEX.

Lipid peroxidation is another key factor of ferroptosis. ACSL4 plays a major role in PL-PUFAs production [69]. Upregulation of ACSL4 increases the PUFA content in phospholipids, leading to enhanced oxidation reactions and ferroptosis [69]. We found that the mRNA expression of ACSL4 significantly increased in neurons isolated from the male TBI + saline group but significantly decreased in microglia isolated from the female TBI + saline group, indicating cell type- and sex-specific lipid metabolism. Decreased ACSL4 can inhibit the production of microglial pro-inflammatory cytokines and lipid peroxidation [70], which can prevent ferroptosis in neurons [24]. Protein levels of 4-HNE and MDA were significantly increased in neurons and microglia in both males and females. DEX treatment significantly reduced 4-HNE and MDA levels in all groups.

Antioxidant defense plays a critical role in maintaining neuronal health and survival against ferroptosis. Evidence indicates that the system X_c_^−^/GSH/GPX4 axis serves to protect against ferroptosis by scavenging PL peroxides [50]. CHAC1 hydrolyzes glutathione, influences calcium signaling and mitochondrial respiratory function, and enhances susceptibility to ferroptosis [71]. We found that TBI significantly decreased GPX4 mRNA expression in microglia, but not in neurons. xCT mRNA expression was significantly decreased in microglia in both male and female TBI + saline groups. In neurons, xCT mRNA expression was significantly reduced only in females, with no significant change observed in males. CHAC1 mRNA expression was significantly increased in both neurons and microglia only in the female TBI + saline group, with no significant changes observed in males. Evidence indicates that expression of ferroptosis genes is cell type-specific, microglia are more sensitive than neurons and astrocytes to ferroptosis [24, 25, 72]. The significantly decreased GPX4 and xCT in microglia may imply that ferroptosis is initiated earlier in microglia, and/or microglia are more susceptible to ferroptosis, in comparison to neurons, especially male neurons. Interestingly, DEX restored GPX4, xCT, and CHAC1 levels in microglia of both males and females but had no significant effect on these markers in neurons from females.

Ferroptosis can propagate intercellularly, sublethal ferroptotic stress triggers microglial inflammatory response that induces neurotoxic astrocytes activation, leading to neuronal death [25]. PTGS2 plays a role in ferroptosis by accelerating arachidonic acid metabolism, releasing inflammatory mediators and activating the inflammatory response [73]. We found that mRNA expression of PTGS2 significantly increased in both neurons and microglia, accompanied by significant upregulation of pro-inflammatory cytokines, such as TNF-α and IL-1β. These results are consistent with our previous study, in which TNF-α and IL-1β rapidly elevated after injury in a cell type and sex specific manner, accompanied with elevated ER stress [20, 74, 75]. TNF-α and IL-1β promote iron influx and simultaneously reduce iron efflux [76]. Evidence indicates that DEX has anti-inflammatory effects [77], we found that DEX effectively decreased pro-inflammatory responses in all groups.

The sex-specific response to ferroptosis and DEX treatment is likely attributable to a combination of factors, including variations in sex hormone levels, differential expression of α2-adrenergic receptors, distinct modulation of inflammatory pathways, differences in blood–brain barrier (BBB) integrity, and variations in the metabolism and pharmacokinetics of DEX [78]. For example, estradiol has been shown to affect the cellular redox state [79], while estrogen can decrease α2-AR mRNA expression levels and α2-AR binding density [80]. The anxiolytic effect of DEX is more effective in female rats than in male rats [81], while DEX exhibits a stronger morphine-sparing effect in controlling postoperative acute pain in male patients than in female patients [82]. Moreover, males and females exhibit distinct inflammatory responses following TBI. DEX administration has been shown to decrease levels of pro-inflammatory cytokines such as TNF-α and IL-6; however, this reduction tends to be more pronounced in females, potentially due to baseline differences in microglial activation. Our group has previously demonstrated sex differences in neuroinflammatory responses, astrocytic reactions to injury, and subsequent injury recovery [41]. Additionally, male animals demonstrate greater increases in BBB permeability compared to females after injury, while females experience less reduction and show better recovery of cortical blood flow, which can potentially affect the metabolism and pharmacokinetics of DEX [78].

The cell type-specific responses to DEX treatment might be due in part to the differential regulation of α2-adrenergic receptors after TBI. Both neurons and microglia express α2-adrenergic receptors [83, 84]. Evidence indicates that the expression of α2-adrenergic receptors is significantly increased in the activated microglia, compared with the resting microglia [85]. In this study, we have demonstrated that the mRNA expression of α2-adrenergic receptors significantly increased in microglia, but not in the neurons. The increased expression of α2-adrenergic receptors in activated microglia can potentially facilitate the effects of DEX. Indeed, numerous studies have shown that DEX can increase microglial anti-inflammatory polarization by modulating signaling pathways, such as ERK1/2, IκB, NLRP3 and PI3K/Akt [77, 86–88], and protects neurons via miR-377-5p/Arc pathway [89]. On the other hand, studies have shown that expression of α2-adrenergic receptors has decreased after TBI [90]. This discrepancy may be attributable to variations in the time points at which α2-adrenergic receptor levels were assessed (e.g., 6 h versus 7 days post-injury). However, additional factors, such as differences in hormone levels, signaling pathway activation, epigenetic modifications, and cellular antioxidant capacities, may also contribute to the sex- and cell type-specific effects of DEX on ferroptosis. Future research is warranted to manipulate specific pathways to fully elucidate the underlying mechanisms responsible for these observed differences.

This study has some limitations. Here, we have only measured the outcomes at 6-h post-injury. The cells’ responses and the efficacy of DEX can be different at the acute, subacute and chronic phases. Therefore, it is worth exploring multiple time points post-injury, which will facilitate the definition of therapeutic time window. Due to the low protein yield from isolated primary neurons and microglia from each animal, most markers related to iron metabolism, oxidative stress, and neuroinflammation were only assessed at the mRNA level. Confirming these findings at the protein level would further strengthen the conclusions. Moreover, α2-adrenergic receptors are expressed both presynaptically and postsynaptically [91, 92]. In our future study, we will differentiate the expression level of the presynaptic and postsynaptic α2-adrenergic receptors.

## Conclusions

In conclusion, TBI results in cell type- and sex-specific activation of ferroptosis pathways in neurons and microglia at the acute phase post-injury in immature brains. DEX administration exhibits cell type- and sex-specific effects on ameliorating ferroptosis in neurons and microglia. To the best of our knowledge, this study is the first to elucidate the cell type- and sex-specific effects of DEX on ferroptosis after TBI in immature brains.

## Supplementary information

Below is the link to the electronic supplementary material.

ESM 1 (DOCX 569 KB)

## Data Availability

The datasets generated and analyzed during the current study are available from the corresponding author upon reasonable request.
