# Supplementary material for: Sex- and cell type-specific effects of dexmedetomidine on ferroptosis in neurons and microglia following traumatic brain injury in juvenile mice
Source: Mol Neurobiol. 2025 Nov 6;63(1):4. doi: 10.1007/s12035-025-05281-x (PMC12592262; doi:10.1007/s12035-025-05281-x)
Supplement: Supplementary file 1 — (DOCX 569 KB) [file 12035_2025_5281_MOESM1_ESM.docx]

**Supplemental Data**

**
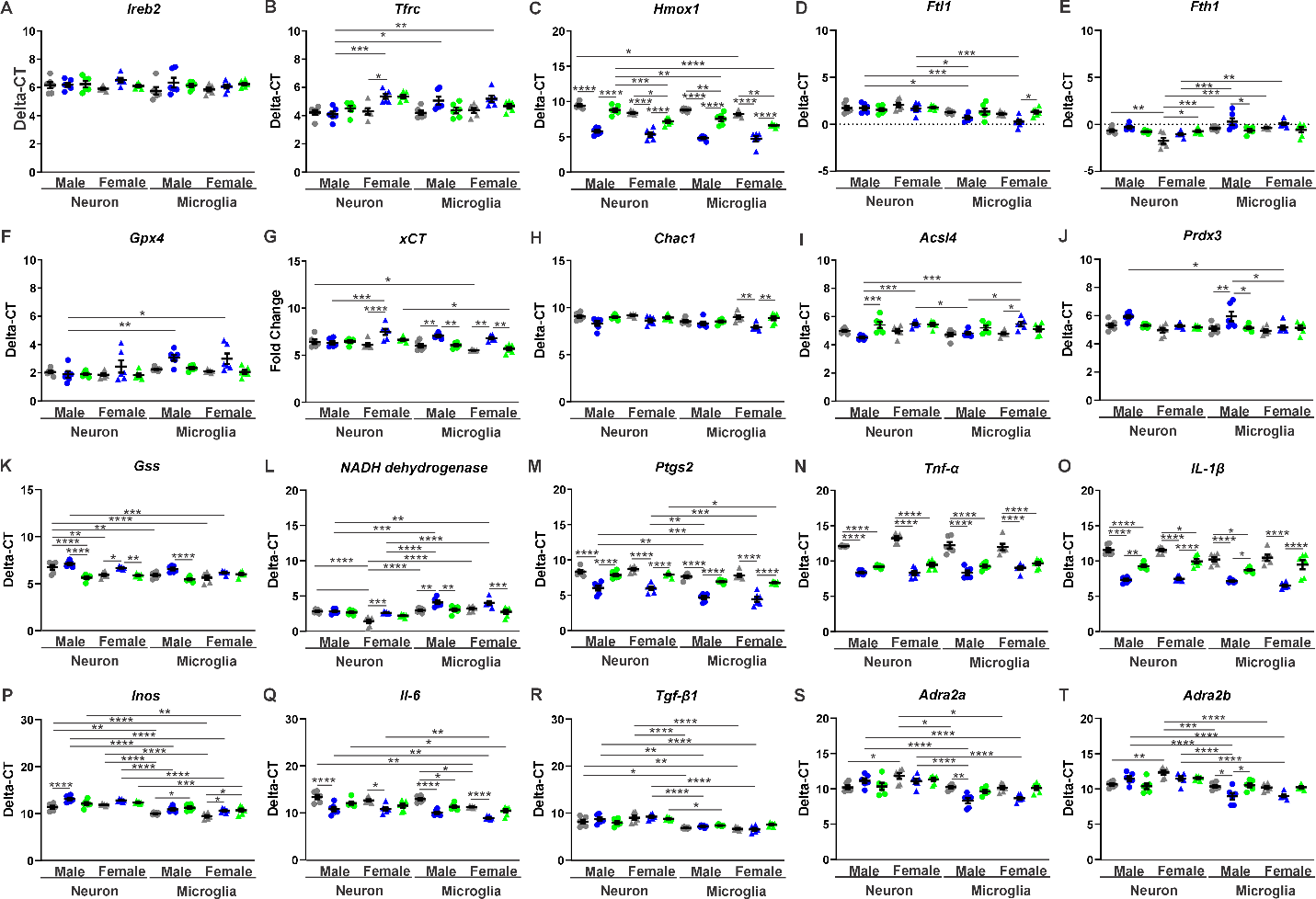
**

**Supplemental Fig. 1** For qPCR analysis, ΔCt values, representing target gene expression normalized to GAPDH, are reported for all experimental groups. Note that lower ΔCt values correspond to higher expression levels of the target gene. Statistical significance is indicated as follows: *p < 0.05; **p < 0.01; ***p < 0.001; ****p < 0.0001.

**
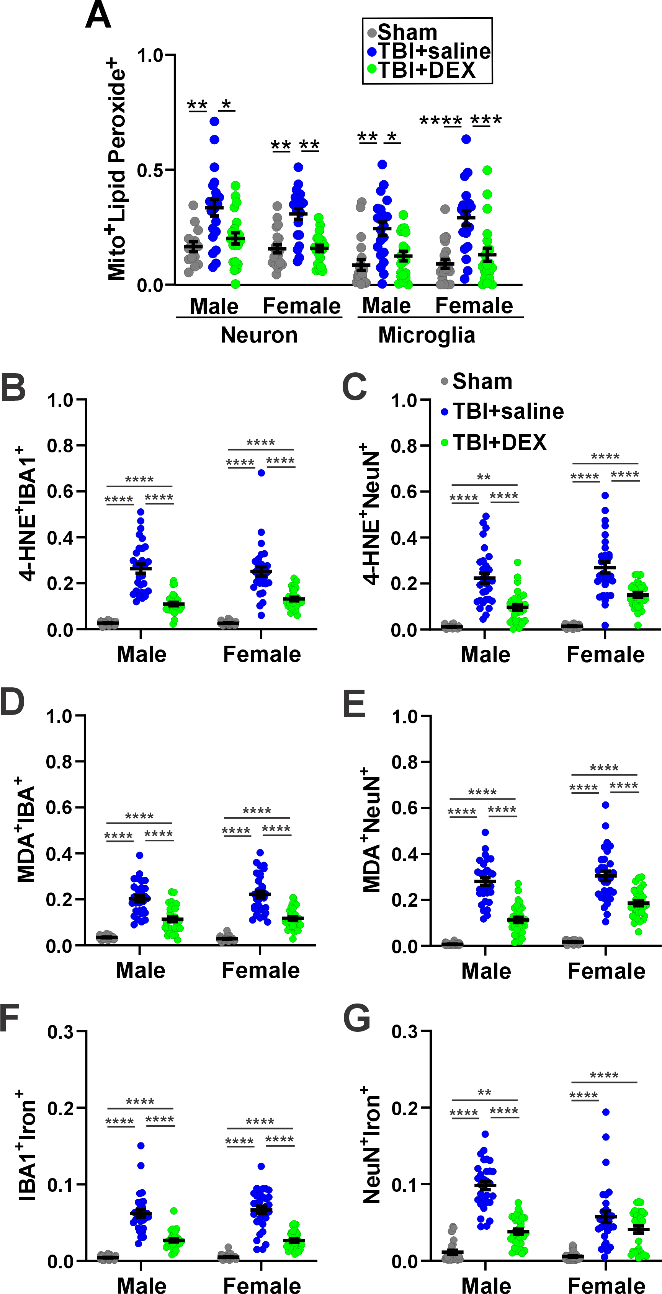
**

**Supplemental Fig. 2** The histological quantification. **A** The co-localization of mitochondria and mitochondrial lipid peroxide significantly increased in the TBI+saline groups, compared with the sham and TBI+DEX groups in both males and females (5-7 images/replicate, 3 replicates per group). **B-G** The co-localization of 4-HNE, MDA, and iron with neurons (NeuN postitive) and microglia (IBA1 positive). 5 images/animal from Sham (n=12; 6M/6F), TBI+saline (n=12; 6M/6F), and TBI+DEX (n=12; 6M/6F). Statistical significance is indicated as follows: *p < 0.05; **p < 0.01; ***p < 0.001; ****p < 0.0001.
